# Eccentric-Overload Production during the Flywheel Squat Exercise in Young Soccer Players: Implications for Injury Prevention

**DOI:** 10.3390/ijerph17103671

**Published:** 2020-05-22

**Authors:** Javier Raya-González, Daniel Castillo, Marta Domínguez-Díez, José Luis Hernández-Davó

**Affiliations:** Facultad de Ciencias de la Salud, Universidad Isabel I, 09001 Burgos, Spain; rayagonzalezjavier@gmail.com (J.R.-G.); mdomid00@gmail.com (M.D.-D.); jlhdez43@gmail.com (J.L.H.-D.)

**Keywords:** iso-inertial devices, resistance training, maximal power output, rotary device, team sports

## Abstract

This study aimed to evaluate the differences in power production between movement phases (i.e., concentric and eccentric) during the execution of resistance exercises with a flywheel device, differentiating between execution regimes (i.e., bilateral, unilateral dominant leg and unilateral non-dominant leg). Twenty young elite soccer players (U−17) performed two sets of six repetitions of the bilateral half-squat (inertia 0.025 kg·m^−2^) and the lateral-squat exercise (inertia 0.010 kg·m^−2^) on a flywheel device. During the testing sessions, mean and peak power in concentric (MPcon) and eccentric (MPecc) phases were recorded. The non-dominant leg showed higher values in all power variables measured, although substantial differences were only found in MPecc (ES = 0.40, likely) and PPcon (ES = 0.36, possibly). On the other hand, for both exercises, MPcon was higher than MPecc (ES = −0.57 to −0.31, possibly/likely greater), while only PPecc was higher than PPcon in the dominant lateral-squat (ES = 0.44, likely). These findings suggest that young soccer players have difficulty in reaching eccentric-overload during flywheel exercises, achieving it only with the dominant leg. Therefore, coaches should propose precise preventive programs based on flywheel devices, attending to the specific characteristics of each limb, as well as managing other variables to elicit eccentric-overload.

## 1. Introduction

Soccer is a team sport characterized by a large number of high intensity and short-term actions which demand the production of unilateral actions such as sprints, changes of direction or jumps [1]. These powerful actions usually precede the most determinant actions in soccer (e.g., goal) [2], whereby improving the strength of the lower limbs seems to be a key strategy to maximize the players’ on-field competitive performance [3,4]. In this regard, some authors have highlighted the relationship between higher power outcomes (i.e., mean and peak power) and the capacity to perform high-intensity actions (i.e., sprint, vertical jump and change of direction) [5]. Thus, the optimization of the ability to generate maximal power in short time-periods is considered crucial not only to improve soccer performance [6,7], but to reduce injury risk [8].

Resistance training is considered the most appropriate method to improve muscular power [9,10]. Traditionally, free weights and weight stack machines have been used [11], which involve a high stimulus during the concentric (CON) phase in contrast with a much lower activation during the eccentric (ECC) phase of the movement [12]. However, ECC actions during resistance programs are characterized by producing higher peaks of force [13] with lower muscle activation [14], lower metabolic cost [15] and earlier increments in muscle mass [16] in relation to CON-based resistance exercises. These advantages, along with the importance of the ECC power to decelerate and stabilize the body during the braking phase in specific soccer actions such as jumps or changes of direction [17], have led to an exponential increase of interest in the study and the use of this type of resistance exercise during the soccer players’ training periodization [16,18].

In the last few years, in order to optimize the load managed during the ECC phase, flywheel devices (FD) have been developed [19]. These devices are characterized by providing resistance through the inertia generated by rotating flywheels [20]. During the CON phase, the force applied unwinds a strap connected to the shaft of the device, which starts to rotate. Once the CON action is completed, the strap rewinds and the athlete must resist the pull of the device by performing a braking, ECC muscle action [21], which generates a substantial eccentric-overload (EO) (i.e., greater ECC than CON force production) [12,22]. In practical terms, the efficacy of the FD in team-sports has been confirmed by enhancing hypertrophy [12,23], jumping ability [24,25], sprint time [24,26], and change of direction ability [23,27] involving athletes in flywheel training programs over 6 and 27 weeks. In addition, this kind of stimulus has been shown to be beneficial in injury prevention programs [12,24] and rehabilitation programs [28] since literature has demonstrated the reduction of injury incidence and the burden values in soccer players. Thus, power training based on the FD constitutes an attractive strategy to implement with soccer players [29], which is being widely used by professional soccer teams [30,31].

Despite the assumption that the use of the FD elicits EO [12,22], there is some controversy about it. Sabido et al. [18] showed a high EO after the completion of the half-squat exercise with an FD using different inertias (i.e., 0.025 kg·m^−2^, 0.050 kg·m^−2^, 0.075 kg·m^−2^ and 0.100 kg·m^−2^) in high-level handball players. Norrbrand et al. [8] also showed greater peak power (PP) values in the ECC phase in relation to the CON phase when healthy men were performing the leg curl exercise using an FD. Nevertheless, Tous-Fajardo et al. (2006) only found EO when training with experienced athletes (e.g., rugby and soccer players) but not with those considered inexperienced. According to the results obtained in the aforementioned studies, numerous variables such as the exercise used (e.g., half squat or leg curl), the level of the participants (i.e., experienced or inexperienced), the inertial load used (i.e., higher inertias produce higher EO) or the power variable chosen for the analysis (i.e., mean power or peak power) must be taken into account during FD preventive training programs.

In this sense, it seems necessary to study if the FD produces EO per se, and if both legs (i.e., dominant and non-dominant) present the same responses to the stimuli generated by the FD. Therefore, the main purpose of this study was to analyze the differences in power production between movement phases (i.e., CON and ECC) attending to different execution regimes (i.e., bilateral, unilateral dominant leg and unilateral non-dominant leg) during the execution of resistance exercises with an FD. We hypothesized that while the mean power (MP) would be higher during the CON phase, the PP would be greater in all regimes during the ECC phase. In addition, we hypothesized that limb-dominance differences would be detected in young professional soccer players.

## 2. Materials and Methods

### 2.1. Participants

Twenty young professional soccer players (age: 16.7 ± 0.3 years, height: 178 ± 6 cm, body mass: 72.1 ± 7.1 kg, and body mass index: 22.1 ± 1.3 kg·m^-^²) belonging to the same U-17 soccer team of the Spanish First Division Club took part in the study (experience in the club = 6 ± 3 years). None of the participants had experience in the use of the FD, although all of them had at least one year’s experience in strength training. During the study, participants were within the competitive phase of the season. The habitual training schedule consisted of four (90 min) training sessions per week. During the period of the study, no resistance training was performed. The inclusion criteria were that the players completed all the familiarization and test sessions and that they had not suffered any injury in the two months prior to the assessment. All the participants were informed of the objectives of the research, participated voluntarily and had the possibility to withdraw at any time from the investigation without any penalty. A written informed parental consent was obtained from each participant who was under 18 years of age, and participants over the age of 18 gave their signed informed consent. The study followed the guidelines set out in the Declaration of Helsinki (2013) and was approved by the Research Ethics Committee of the Universidad Isabel I (Code: PI−008).

### 2.2. Experimental Design

**Figure 1:**
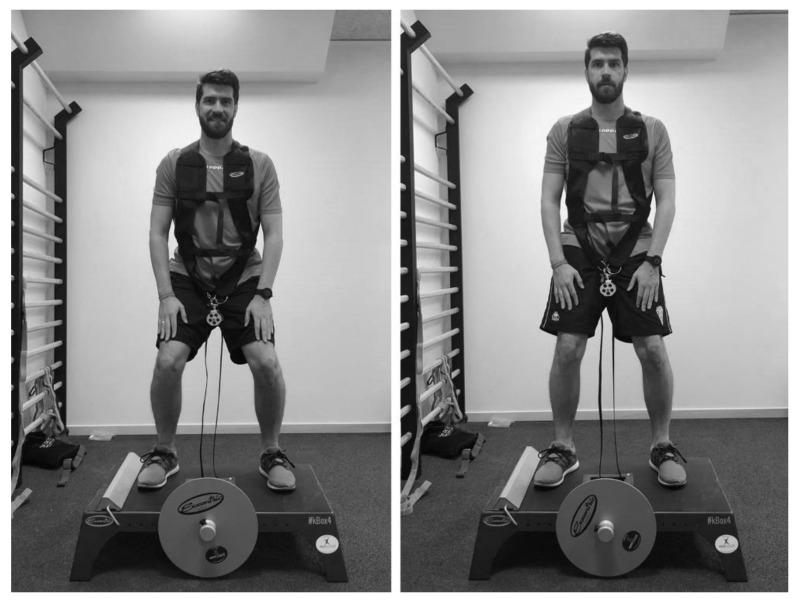
Half-squat exercise performed in the study.

**Figure 2:**
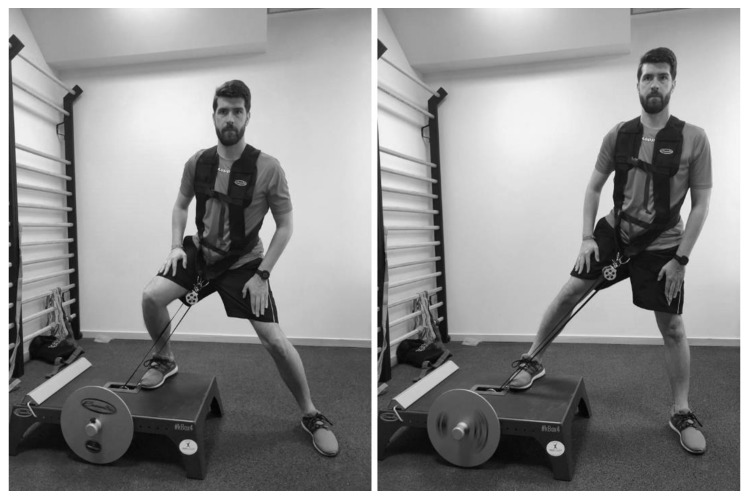
Lateral-squat exercise performed in the study.

This research analyzed the power output produced during the execution of two flywheel-resistance exercises (i.e., half-squat and lateral-squat) in young soccer players (Figure 1). During the first week, participants were subjected to three familiarization sessions, in which the technique of both exercises was explained and trial sets were carried out [24]. In the second week, players performed a test to select the inertia to be used during the power assessment in the flywheel devices (i.e., the inertia that achieved higher power output) [24]. A power test was developed 72 h later, to know the power values (i.e., MP and PP in both CON and ECC phases) of both legs (i.e., dominant and non-dominant) during flywheel-resistance exercise execution (Figure 1 and Figure 2). The dominant leg was defined as the leg that the soccer player primarily prefers to use when kicking the ball and the non-dominant leg was defined as the leg that the soccer player uses to perform support when kicking the ball [32]. Participants were asked to refrain from intense exercise for at least 48 h before any testing session and not to consume any food or caffeine in the 3 hours prior to any test [25]. Prior to both assessment sessions (i.e., inertial selection and power assessment) a standardized 15 min warm-up, including cycling, joint mobility, dynamic stretching and bilateral and unilateral jumps, as well as a submaximal set of eight repetitions of the half-squat flywheel exercise, was performed.

### 2.3. Power Assessment

An assessment with four different inertias (4 repetitions per inertia with 240 s inter-inertia recovery) was performed to determine the inertia during the power test. The inertia that achieved higher mean power output was selected (i.e., half-squat = inertia 0.025 kg·m^−2^ and lateral-squat = inertia 0.010 kg·m^−2^). The following protocol was performed using a specific analysis feature in a performance-measurement system compatible with flywheel devices (SmartCoach™ Power Encoder, SmartCoach Europe AB, Stockholm, Sweden) with the associated software (SmartCoach software, Stockholm, Sweden, v5.6.0.8).

The power test was developed using an FD (K-Box 4, Exxentric, Stockholm, Sweden) by means of a rotary encoder (SmartCoach Power Encoder, SmartCoach Europe AB, Stockholm, Sweden). All players performed randomly 2 sets of 6 repetitions of the half-squat (inertia 0.025 kg·m^−2^) and 2 sets of 6 repetitions of the lateral-squat exercise with each leg (inertia 0.010 kg·m^−2^), with a rest of 4 min between each set. After two initial submaximal repetitions to initiate the flywheel movement, participants were encouraged to perform the CON phase (i.e., from 90° knee flexion to full extension) as fast as possible. Power parameters (i.e., MPcon, MPecc, PPcon and PPecc) were measured in each exercise and used for the subsequent statistical analysis, using the best result obtained in each test. To ensure the proper technique, all the executions were supervised by a strength and conditioning coach and a mirror was placed opposite to the participants to let them visually check their technique [33].

### 2.4. Statistical Analyses

Results are presented as mean ± standard deviations (SD). Relative reproducibility of the data was performed using the intraclass correlation coefficient (ICC) for each power variable [34]. Effect sizes (ES) with the uncertainty of the estimates shown as 90% confidence limits (CL) were used to quantify the magnitude of the difference between legs and movement phases (i.e., CON and ECC). The ES were classified as trivial (< 0.2), small (0.2–0.6), moderate (0.6–1.2), large (1.2–2.0), very large (2.0–4.0) and extremely large (> 4.0) [34]. These changes were then qualified via probabilistic terms and assigned using the following scale: 25%–75%, possibly; 75%–95%, likely; 95%–99.5%, very likely; >99.5%, most likely [34]. Inference was classified as unclear if the 90% CL overlapped the thresholds for the smallest worthwhile positive and negative effects [34]. The mean differences, CL, ES and magnitude-based inferences were calculated using a specific spreadsheet (post-only crossover) available at www.sportsci.org [35].

## 3. Results

The reliability inter-subjects showed high values for each power variable as revealed by the ICC, in half-squat (ICC = 0.83 [0.78–0.88]), dominant leg lateral-squat (ICC = 0.85 [0.83–0.87]) and non-dominant leg lateral-squat (ICC = 0.83 [0.78–0.87]).

Table 1 shows the mean difference in power variables between limbs along with ES and qualitative inferences. Lower values were found when comparing the dominant with the non-dominant leg for MPecc (likely small; ES = 0.40) and PPcon (possibly small; ES = 0.36).

**Figure 3:**
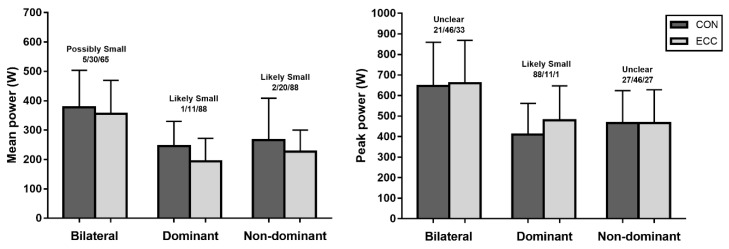
Practical differences in power variables between movement phases.

The power values registered in CON and ECC phases in each execution regime are presented in Figure 3. Higher MP values were found in CON than in ECC phases in both dominant and non-dominant legs (likely small; ES = −0.57 ± 0.52 and −0.43 ± 0.50, respectively) but possible differences were found in bilateral execution. However, greater PPecc was observed in the dominant leg (likely small; ES = 0.44 ± 0.57).

## 4. Discussion

This study aimed to analyze the differences in power production between movement phases (i.e., CON and ECC) during the execution of resistance exercises with an FD, differentiating between execution regimes (i.e., bilateral, unilateral dominant and non-dominant leg). Although previous studies have focused on knowing the EO produced by an FD, this is the first investigation that statistically analyses the differences in power obtained in both movement phases with young soccer players. The main results of the study showed that lower values of MPecc and PPcon were found when comparing the dominant with the non-dominant leg. In addition, greater MP values were found in the CON than in the ECC phase in both the dominant and the non-dominant leg, while greater PPecc compared to PPcon was observed only in the dominant leg.

In soccer, due to the high intensity of short-term actions reflected in the high demands on unilateral force production, higher power values have been previously shown in the non-dominant leg in relation to the dominant leg [36,37]. This higher power production performed by the non-dominant leg has been explained by leg specialization: the dominant leg is used to manipulate the ball (during kicking, dribbling or passing) and the non-dominant leg performs the support and stability functions of the body itself [38]. However, in the present study the values of the power variables found in the non-dominant leg were not substantially greater than in the dominant leg (Table 1), and substantial differences (small) were observed in MPecc and PPcon. These small differences could be due to the lower number of years of systematized training in young soccer players. Despite this, bilateral differences must be considered due to their relationship with the increase of the injury risk [39]. In this sense, it would be interesting to know the fitness level in relation to strength/power values of each limb, with the objective to formulate precise preventive training programs based on the FD, which could lead to a reduction in bilateral asymmetries [40].

EO has been calculated as the difference between ECC and CON power [41] and it is considered that there is an EO when the ECC power value is greater than the CON one. In this sense, some previous studies with handball players [42,43] have shown higher values in PP in the ECC phase than in the CON phase during flywheel half-squats, while other authors [12,44] have not found EO with healthy men and inexperienced rugby and soccer players during the flywheel leg curl. In the current study, the results obtained seem to be similar to the aforementioned studies in which no EO was obtained [12,44]. In this sense, greater power values were not found in the ECC phase compared to the CON phase, and even higher values of mean power during the CON phase were observed during unilateral executions (i.e., dominant leg and non-dominant leg). These results show the need to apply different strategies (e.g., to provide instructions that encourage the participants to delay the braking action to the last third of the ECC phase) when the EO is a target of the preventive training programs [41]. On the other hand, the PP during the ECC phase was substantially higher in the dominant leg in comparison to the CON phase (likely small). This could be due to this leg being accustomed to brusque ECC actions (e.g., the moment of kicking the ball), generating EO due to a better braking-skill than the non-dominant leg. This disability of the non-dominant leg to produce EO is an aspect to consider when programming preventive training protocols with young soccer players, since the EO influences the effects of training with FD [45]. In this regard, the aforementioned strategies should be implemented mainly when FD exercises are executed with this leg. Additionally, load variables (i.e., volume, intensity and recovery) should be managed in a differentiated way according to each leg.

This study is not exempt of limitations. The main one is that only the power values were assessed. In this sense, similar further studies using power plates to know the differences in force would be necessary. Another limitation is the sample demographic (i.e., young male soccer players), so the possibility of extrapolating these results to other populations is limited. For further studies, it would be relevant to include male and female soccer players of different ages and levels. Finally, considering that only the half squat and lateral squat exercises were performed, future studies could assess the power outcomes related to other specific movements for soccer practice (i.e., horizontal axis) executed on FD.

## 5. Conclusions

Execution regimes (i.e., dominant or non-dominant) influence the power generated during resistance exercises executed on an FD by young soccer players in each movement phase (i.e., CON and ECC), showing greater MPecc values in the non-dominant leg in comparison to the dominant leg. These findings suggest coaches should propose concrete FD preventive programs attending to the specific characteristics of each limb in order to reduce bilateral asymmetries. Additionally, these results have shown that FD do not produce EO in dominant and non-dominant limbs in young players, except in the PP of the dominant leg, possibly due to the specific features of this leg in soccer players. Considering that the players did not achieve EO using the FD, strength and conditioning coaches should manage different variables (e.g., inertial load used, movement pattern technique, instructions) aiming to reach EO during FD exercises, which has been previously shown as a determinant factor for strength adaptation.

## Figures and Tables

**Table 1:** Mean differences ± SD in power variables among dominant and non-dominant limbs along with effect sizes (ES) and qualitative inferences.

Variables	Dominant Leg	Non-Dominant Leg	Mean Difference; ± 90% CL	ES; ± 90% CL	Qualitative Inference	Rating
MPcon (W)	244.89 ± 85.04	265.62 ± 87.43	9.1; ±24.8	0.23; ± 0.55	Unclear	54/37/9
MPecc (W)	194.18 ± 77.72	226.44 ± 73.07	19.2; ±27.7	0.40; ± 0.52	Likely Small	75/23/2
PPcon (W)	410.33 ± 151.26	467.08 ± 156.83	14.8; ± 26.6	0.36; ± 0.55	Possibly Small	69/27/5
PPecc (W)	466.58 ± 161.26	479.69 ± 167.48	2.1; ± 23.0	0.07; ± 0.53	Unclear	35/46/19

Abbreviations: CL = confidence limits; SD = standard deviation; MPcon = concentric mean power; MPecc = eccentric mean power; PPcon = concentric peak power; PPecc = eccentric peak power.
